# Perspectives and Insights into the Competition for Aminoacyl-tRNAs between the Translational Machinery and for tRNA Dependent Non-Ribosomal Peptide Bond Formation

**DOI:** 10.3390/life6010002

**Published:** 2015-12-31

**Authors:** Angela W. S. Fung, Roshani Payoe, Richard P. Fahlman

**Affiliations:** 1 Department of Biochemistry, Faculty of Medicine & Dentistry, University of Alberta, 474-MSB Edmonton, AB T6G 2H7, Canada; awfung@ualberta.ca (A.W.S.F.); payoe@ualberta.ca (R.P.); 2 Department of Laboratory Medicine and Pathobiology, Faculty of Medicine, University of Toronto, Toronto, ON M5S 1A1, Canada; 3 Institute of Technology, Faculty of Science and Technology, University of Tartu, Noorse St 1, Tartu 50411, Estonia; 4 Department of Oncology, Faculty of Medicine & Dentistry, University of Alberta, Edmonton, AB T6G 2H7, Canada

**Keywords:** tRNA, aminoacyl-tRNA, L/F-transferase, stringent response, EF-Tu, N-end rule, (p)ppGpp

## Abstract

Aminoacyl-tRNA protein transferases catalyze the transfer of amino acids from aminoacyl-tRNAs to polypeptide substrates. Different forms of these enzymes are found in the different kingdoms of life and have been identified to be central to a wide variety of cellular processes. L/F-transferase is the sole member of this class of enzyme found in *Escherichia coli* and catalyzes the transfer of leucine to the N-termini of proteins which result in the targeted degradation of the modified protein. Recent investigations on the tRNA specificity of L/F-transferase have revealed the unique recognition nucleotides for a preferred Leu-tRNA^Leu^ isoacceptor substrate. In addition to discussing this tRNA selectivity by L/F-transferase, we present and discuss a hypothesis and its implications regarding the apparent competition for this aminoacyl-tRNA between L/F-transferase and the translational machinery. Our discussion reveals a hypothetical involvement of the bacterial stringent response that occurs upon amino acid limitation as a potential cellular event that may reduce this competition and provide the opportunity for L/F-transferase to readily increase its access to the pool of aminoacylated tRNA substrates.

## 1. Introduction

The evolutionary sequence diversity of tRNAs is constrained by the selective pressures to maintain key nucleotides recognized by a variety of cellular factors, such as aminoacyl-tRNA synthetases, translation factors and a variety of other factors that utilize tRNAs for functions beyond that of protein synthesis. These cellular processes include both ribosome-dependent and ribosome-independent peptide bond formation reactions in addition to other cellular processes [1]. Perhaps the only examples where the biological role of tRNAs do not present evolutionary constraints are the role of a host’s tRNA acting as primers for reverse transcriptases in retroviruses and retrotransposons [2].

It has become increasingly clear that the translational machinery is not the only cellular system that utilizes aminoacyl-tRNAs as substrates. Specific aminoacyl-tRNAs have been demonstrated to serve as a source of activated amino acids in tRNA-dependent ribosome-independent peptide bond formations in processes such as peptidoglycan biosynthesis [3,4], cellular membrane remodeling [5,6,7], antibiotic biosynthesis [8,9], and targeted proteolysis [10]. The utilization of aminoacyl-tRNA as substrates by the enzymes central to these functions results in the potential for the competition for tRNA substrates between the translational machinery and ribosome-independent peptide bond formation reactions. 

A variety of mechanisms have been proposed for how these aminoacyl-tRNAs evade the translation machinery, for example, in some gram-positive eubacteria, aminoacyl-tRNAs are substrates for the synthesis of the interpeptide linkers of cell wall peptidoglycan biosynthesis [3,4]. In *Staphylococcus aureus*, a specialized tRNA^Gly^ isoacceptor which appears to not be efficiently used by the translational machinery but is preferentially utilized for peptidoglycan biosynthesis [11]. The development of specialized tRNA isoacceptors may be a mechanism to prevent a detrimental competition for substrates between translation and cell wall biosynthesis, both highly active processes during cell growth. Other examples of evasion mechanisms include limiting access to the aminoacyl-tRNAs by channeling the substrates through enzyme complexes with aminoacyl-tRNA synthetases [9], or having comparable affinities for aminoacyl-tRNA such that there is sufficient competition with elongation factor EF-Tu [5].

tRNA-dependent ribosome-independent peptide bond formation is catalyzed by a family of enzymes called aminoacyl-tRNA protein transferases, which are expressed in very diverse organisms and are involved in N-end rule protein degradation pathways. Aminoacyl-tRNA protein transferases are a class of enzymes that transfer amino acids from an aminoacyl-tRNA to the N-terminus of a protein, which either marks it for degradation by the cellular machinery or alters the proteins’ function. These enzymes are found in eubacteria [12], yeast [13], plants [14], and animals [15]. It has been demonstrated that aminoacyl-tRNA protein transferases in eukaryotes have a large range of physiological functions *in vivo*, including heart development [16], G-protein signalling [17,18], gametogenesis [19], and apoptosis [20,21,22,23,24] (see review for more details [25]). 

## 2. L/F-Transferase and Its Biological Functions

**Figure 1:**
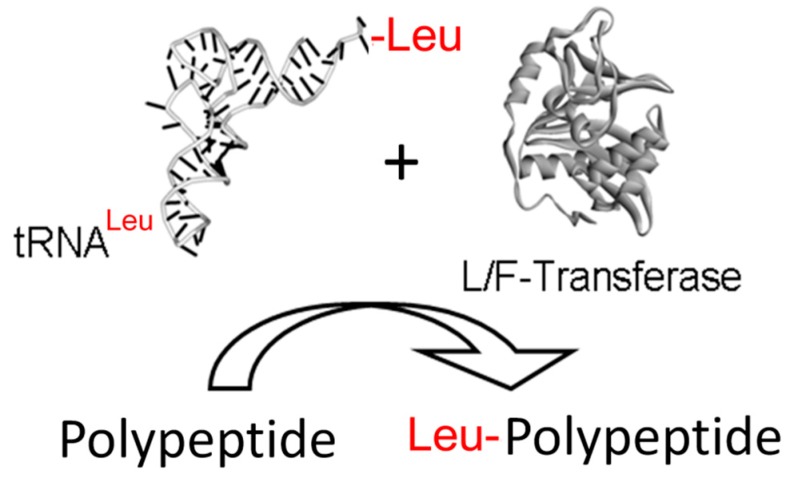
L/F-transferase reaction. A schematic diagram depicting the transfer of an amino acid from an aminoacyl-tRNA substrate to the N-termini of a polypeptide substrate by L/F-transferase.

In *Escherichia coli*, L/F-transferase is the sole aminoacyl-tRNA-protein transferase [26,27]. Specifically, L/F-transferase catalyzes the transfer of an amino acid from an aminoacyl-tRNA to the N-terminus of a protein (Figure 1) having an N-terminal basic residue (Arg/Lys). This enzyme catalyzes a substrate-assisted peptide bond formation reaction mechanism that is analogous to that proposed for the ribosome [28]. 

Currently, there is only a minimal understanding on the physiological function of N-end rule in prokaryotes, which has been more elusive since only a couple of substrates have been verified to date. Roles in putrescine homeostasis, proline catabolism, peptide transport, growth phase-dependent proteolysis and stationary phase exit have been proposed [27,29,30,31]. 

To date, only two *in vivo* protein substrates of L/F transferase have been identified and validated, and they are putrescine aminotransferase (PATase) and DNA protection during starvation protein (Dps) [30]. PATase catalyzes the aminotransferase reaction to convert putrescine to 2-oxoglutarate to generate L-glutamate and 4-aminobutanal, where putrescine is a polyamine and polyamines have been shown to also be involved in protein biosynthesis, oxidative stress and biofilm formation [32,33,34]. The degradation of PATase via the N-end rule may be a mechanism to ensure putrescine homeostasis. Dps plays a role in protecting DNA during starvation and oxidative stress by forming a complex with DNA or by chelating iron from toxic by-products of the Fenton reaction [35]. In bacteria, Dps levels are low during exponential growth but increase upon starvation and oxidative stress [36]. Dps levels have been demonstrated to be dependent on the growth phase by two separate mechanisms, via a ClpXP-dependent or an L/F-transferase-dependent targeted proteolysis [30,31,36,37,38]. 

Recently, a more thorough study identified over 100 putative *E. coli* N-end rule substrates, many of which belong to large protein complexes suggesting roles in remodelling or regulation of protein complexes [39]. Contrary to earlier understanding, there seems to be no correlation between L/F-transferase-dependent proteolysis and different growth phases since the authors observed that certain N-end rule substrates are enriched during exponential growth while others are enriched during the stationary phase. These observations and identified putative substrates of *E. coli* L/F-transferase and N-end rule remain to be further tested and validated. If more evidence is provided to relate L/F-transferase’s role in regulating these putative substrates, it has been suggested that the *E. coli* N-end rule may play a more central role in biological processes than previously recognized including but not limited to cell division, DNA replication, transcription, translation, metabolism, as well as protein quality control [39]. 

## 3. Substrate Recognition by L/F-Transferase

### 3.1. Sequence Recognition of Protein Substrates by L/F-Transferase

While the focus of this review is on tRNA recognition, there is also a growing knowledge regarding protein substrate recognition. Shortly after the discovery of L/F-transferase activity it was demonstrated that it exhibited selectivity for protein substrates with basic N-termini [40]. Since this earlier work, the identification of the first *in vivo* substrate of L/F-transferase revealed the transfer of and amino acid to the N-termini of a protein with an N-terminal methionine [30]. Recent investigations have also identified that the identity of the penultimate amino acid also influences L/F-transferase recognition [41]. For a more detailed discussion regarding the recent discoveries regarding protein substrate recognition, the reader is directed to a recent review [42].

### 3.2. tRNA Recognition by L/F-Transferase

While studies have demonstrated that L/F-transferase can utilize a number of different aminoacyl-tRNA substrates *in vitro* such as tRNA^Leu^, tRNA^Phe^ and tRNA^Met^ [26], *in vivo* investigations indicate that tRNA^Leu^ is the natural substrate [27]. The majority of recognition and specificity appears to arise from the recognition of RNA components of the aminoacyl-tRNAs as the inhibition of L/F-transferase by the aminoacyl-tRNA analogue puromycin (or another related compound) is minimal in comparison to the Km of aminoacyl-tRNA utilization by the enzyme [43]. In addition, a number of investigations have demonstrated the *in vitro* utilization of tRNAs aminoacylated with a variety of unnatural amino acids of varying sizes [44,45,46,47]. Investigations with mis-charging different tRNAs with different amino acids have revealed the major selectivity for the amino acid on an aminoacyl-tRNA substrate is the exclusion of C-beta branched amino acids [48]. This broad specificity for amino acid side chains is the basis for the proposed utilization of L/F-transferase for enzymatic labeling of polypeptides [45] for the rapid synthesis of reagents for applications such as positron emission tomography (PET) imaging [49].

**Figure 2:**
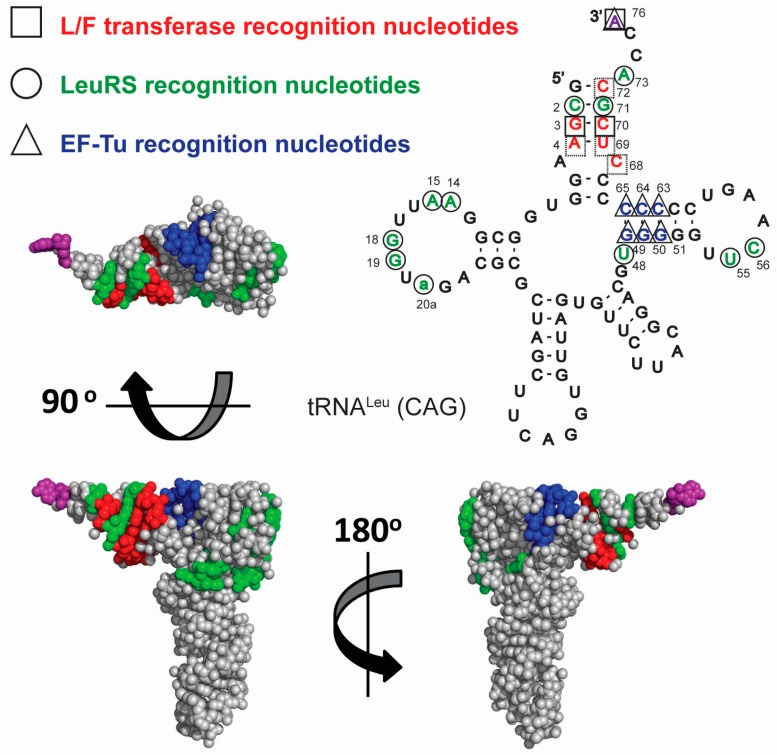
Cloverleaf and 3D (sphere representation) model structure of tRNA^Leu^ (CAG) with key recognition nucleotides and their respective 3D (sphere-representation) surfaces recognized by L/F-transferase (red □), LeuRS (green ○), and EF-Tu (blue ∆) highlighted. The 3'-aminoacyl adenosine (A76) is highlighted in purple to indicate that it is required to be recognized by both L/F-transferase and EF-Tu. The 3D model was generated using PDB ID 1EHZ and PyMOL version 1.41, and nucleotide numbering is according to [52].

Recent investigations into the tRNA sequence specificity by L/F-transferase has confirmed the higher selectivity for tRNA^Leu^ but also preferential selectivity for a single tRNA^Leu^ isoacceptor (anticodon 5′-CAG-3'), as has been previously suggested [48,50,51]. The step-wise mutagenesis of a poor tRNA^Leu^ substrate (with an 5'-GAG-3' anticodon) to that of the optimal tRNA^Leu^ substrate (with a 5'-CAG-3' anticodon) identified key recognition nucleotides for L/F-transferase [50]. Figure 2 shows the cloverleaf sequence and structure of *E. coli* tRNA^Leu^ (CAG) and a 3D model structure using tRNA^Phe^ (since there is no intact complex-free tRNA^Leu^ X-ray crystal structure solved to date, PDB ID: 1EHZ). Key nucleotides recognized by L/F-transferase (red, squares), LeuRS (green, circles) and EF-Tu (blue, triangles) are highlighted. Of particular interest is the lack of overlap between key recognition nucleotides by L/F-transferase and the key recognition nucleotides by the translational machinery (leucyl-tRNA synthetase (LeuRS) or EF-Tu). Although L/F-transferase and EF-Tu both require the recognition of 3' aminoacyl-A76 (purple), they appear to be recognizing different faces of the acceptor stem/T-stem of the tRNA molecule. Meanwhile, LeuRS recognizes both the acceptor stem as well as the tertiary structure of the tRNA (D-/T-loop interactions). The lack of overlap suggests that there is an additional evolutionary constraint on this tRNA^Leu^ isoacceptor (5'-CAG-3') beyond that dictated by the translational machinery.

### 3.3. tRNA Availability

As opposed to the previously mentioned example with cell wall biosynthesis in *S. aureus*, the most optimal tRNA substrate (isoacceptor 5'-CAG-3') for L/F-transferase is also the most abundant leucyl-tRNA isoacceptor in *E. coli* which decodes the most frequently used codon (5'-CUG-3') in a variety of growth rates and media conditions [53,54,55]. Thus, this optimal tRNA substrate for L/F-transferase is not specific for tRNA-dependent ribosome-independent peptide bond formation but is also significantly used by the translational machinery. This results in what appears to be a direct competition for this tRNA between EF-Tu and L/F-transferase. With the much higher abundance of EF-Tu (~100 µM) and its significantly higher affinity for aminoacyl-tRNAs (K_D_ of ~5 nM) compared to the low *in vivo* concentration and lower affinity for L/F-transferase (~0.5 µM and a K_D_ of ~200 nM) [26,27,56], an apparent conundrum exists with regard to how L/F-transferase can compete for an aminoacyl-tRNA substrate. It has been predicted that EF-Tu is in excess of all cellular pools of aminoacyl-tRNAs, suggesting that no excess aminoacyl-tRNAs are available for L/F-transferase and a direct competition of tRNA substrates exists between the two systems. To overcome the competition with the translational machinery, EF-Tu binding to aminoacyl-tRNAs may have to be inactivated under specific cellular conditions, such that aminoacyl-tRNAs may become available for L/F-transferas, such as conditions of nutrient limitation during the stringent response. Alternatively, other mechanisms must exist for which L/F-transferase obtains aminoacyl-tRNA substrates such as specific interaction with the aminoacyl tRNA synthetase, which could potentially enable a direct exchange to the tRNA to L/F-transferase or the role of yet unknown cellular factors that could assist in aminoacyl-tRNA binding by L/F-transferase.

## 4. Bacterial Stringent Response

The bacterial stringent response is an adaptative mechanism mounted in response to various stress stimuli. The best described example is amino acid limitation by which it was first discovered [57] with the notable appearance of two signalling alarmones penta-phosphate guanosine and tetraphosphate guanosine, collectively referred to as (p)ppGpp [58,59].

RelA/SpoT Homologue (RSH) proteins modulate the intracellular concentration of these alarmones [60]. During nutrient rich growth conditions, tRNAs predominantly exist in an energy rich aminoacylated form in a ternary complex with EF-Tu:GTP, thereby providing a constant supply of amino acid available for protein synthesis by the ribosomes. Depletion of cytosolic amino acids pools consequently affect aminoacylation of tRNAs, leading to the accumulation of deacylated tRNA [61,62]. When present in significant excess over the aminoacylated analogue, deacyl-tRNAs bind to the vacant A-sites on the stalled 70S ribosomes and trap the ribosomes in a state referred to as the RAC or RelA activating complex. RelA upon direct sensing of the A-site bound deacylated tRNA present at the ribosomal A-site synthesizes (p)ppGpp [62,63] which then appears to amplify the signal through a positive allosteric feedback cycle [64]. Elevated intracellular concentration of (p)ppGpp mediates global alteration of metabolism and transcription to circumvent any deleterious effect of the stress conditions and can activate virulence genes of pathogens [65]. The stringent response signalling then appears to be attenuated by passive tRNA dissociation from the ribosomal A-Site [66,67,68].

### 4.1. Stringent Response and the Inactivation of EF-Tu and Translation

Much of the altered gene expression induced by the stringent response is via changes in transcription, where elevated levels of (p)ppGpp directly bind to RNA polymerase [69,70] and DNA primase [71] and FtsZ [72]. It has also been determined that the stringent response also alters protein translation by inhibiting translational GTPases including initiation factor IF-2 [73,74], elongation factors EF-Tu [75] and EF-G [74,75,76].

**Figure 3:**
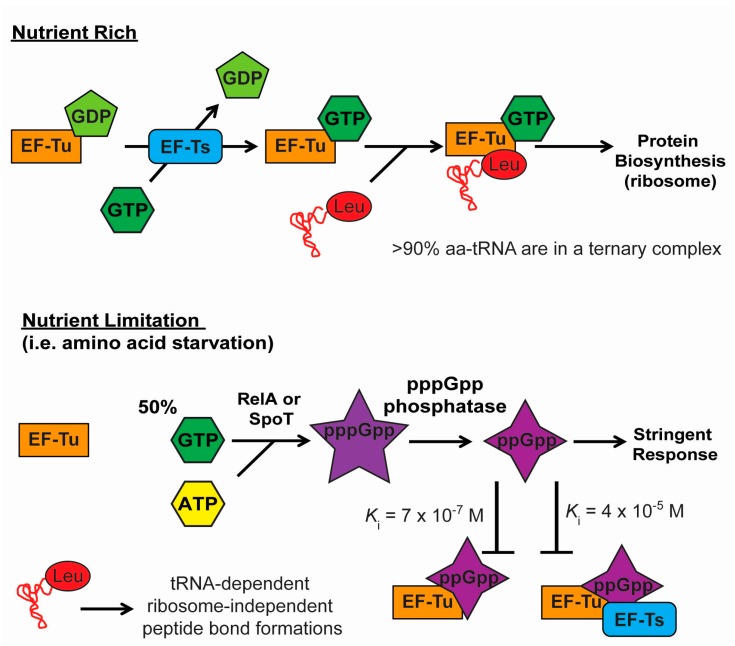
A proposed mechanism for tRNA-dependent ribosome-independent peptide bond formation where aminoacyl-tRNA evades from the translational machinery during the stringent response. During nutrient rich conditions, EF-Tu:GDP is rapidly released and exchanged to the GTP-bound state by the guanine nucleotide exchange factor EF-Ts. EF-Tu:GTP then forms a ternary complex with an aminoacyl-tRNA, and delivers the aminoacyl-tRNA to the ribosomal A-site for protein biosynthesis. During nutrient limiting conditions (*i.e.*, amino acid starvation), GTP molecules are converted to form (p)ppGpp molecules, which results in less GTP molecules available to form the ternary complex, and, thus, EF-Tu cannot bind to aminoacyl-tRNA effectively. ppGpp molecules have also been shown to inhibit EF-Tu and traps EF-Tu in inactive complexes with EF-Ts or ribosomes. Additionally, potential nutrient-dependent post-translational modifications of EF-Tu may further abolishe tRNA binding, while tRNA^Leu^ (CAG) aminoacylation levels are maintained despite amino acid limited conditions. Together, tRNA^Leu^ (CAG) becomes more available for alternative processes such as the L/F-transferase mediated targeted proteolysis.

Figure 3 shows a schematic proposed mechanism where aminoacyl-tRNA may evade the translational machinery during the stringent response such that it becomes available for other cellular processes such as the L/F transferase mediated targeted proteolysis. During the stringent response, approximately half of the GTP molecules are converted to pentaphosphate guanosine (pppGpp) by RelA and/or SpoT, which are further hydrolyzed by guanosine pentaphosphate phosphatases into tetraphosphate guanosine (ppGpp), the functional molecule of the stringent response [77]. Since EF-Tu requires a GTP molecule to form a ternary complex with aminoacyl-tRNA, the loss of a large number of GTP molecules impairs EF-Tu’s ability to bind to aminoacyl-tRNAs efficiently. It has been shown that pppGpp can substitute GTP and binds to EF-Tu to form the ternary complex with aa-tRNA; however, ppGpp mimics the GDP-bound state of EF-Tu such that EF-Tu:ppGpp cannot form the ternary complex with aminoacyl-tRNAs (*K*_i_ = 7 × 10^−7^ M) [78]. The presence of the resultant replacement of GDP with ppGpp further leads to an inactive EF-Tu:EF-Ts:ppGpp complex (*K*_i_ = 4 × 10^−5^ M) [75]. This would effectively trap and inactivate EF-Tu from binding to free aminoacyl-tRNAs, which allows L/F-transferase to bind to free aminoacyl-tRNA for its reaction. Similar tRNA binding abolishing effects by ppGpp have also been observed in initiation factor IF-2 and elongation factor EF-G [73,74].

In addition to the activity of ppGpp, the nutrient status-related post-translational modifications of EF-Tu have also been reported to inhibit its activity. Soares *et al.* showed that, during later phases of growth, the amounts of ribosomal proteins (*i.e.*, S1, S2, S7, L7/L12, L9, L19) and elongation factors (Tu, Ts, G) decrease while their phosphorylation levels increase, suggesting phosphorylation may be important in the regulation of translation in several bacterial species [79]. For example, *E. coli* phosphorylation of EF-Tu at Thr-382 by ribosome-associated kinases has been shown to abolish aminoacyl-tRNA binding, while other bacterial species such as *Mycobecterium tuberculosis* and *Bacillus subtilis* phosphorylation of EF-Tu at various sites have been shown to trap EF-Tu in an inactive EF-Tu:EF-Ts complex, reduce EF-Tu affinity and interaction with GTP, and impair EF-Tu’s GTPase activity, which prevents it from dissociating from the ribosome [80,81,82]. Meanwhile the methylation status of EF-Tu may also play a role in modulating the activity of EF-Tu, as monomethylation at Lys-56 during logarithmic growth and dimethylation at Lys-56 during the stationary phase growth have been observed [83,84].

### 4.2. Stringent Response and Selective Aminoacylation of tRNA Isoacceptors

The rate of codon translation depends on a variety of factors, but the most important factor perhaps is the concentration and charged fractions of the aminoacyl-tRNA isoacceptor with the respective anticodon that reads the codon [55]. It has been estimated that during exponential growth, the charged fractions of all tRNAs are about 80% such that it is sufficient to maintain the rate of translation [85]. However, theoretical modeling and experimental measurement using microarrays and Northern blots analysis show that amino acid limitation results in the selective de-acylation of tRNA, where the charged fractions of some isoacceptors will be low while some will be high [86,87]. Following amino acid limitation, the charged levels of tRNA are expected to first decrease within a few seconds as the cellular pools of aminoacyl-tRNAs turnover rapidly, and are expected to increase over a time period of about 10–50 min as the bacteria begins to biosynthesize amino acids and re-establish the new steady-state values [88]. Specifically, during leucine starvation (2–45 min) in RelA+ auxotroph strain CP78, the aminoacylation levels of tRNA^Leu^ isoacceptors have been measured and categorized: tRNA^Leu^ (CAA) and (UAA) isoacceptors are insensitive to amino acid starvation with aminoacylation levels of 10%–30%, tRNA^Leu^ (CAG) isoacceptor (which is the best substrate for L/F-transferase) is categorized as intermediately sensitive to starvation with an aminoacylation level of 8%, and tRNA^Leu^ (GAG) and (UAG) isoacceptors are sensitive to starvation with an aminoacylation level of 2%–4% [86]. 

## 5. Hypothesis: Is the Stringent Response the Key to the Function of L/F-Transferase?

When considering the inhibition of EF-Tu and overall translation by (p)ppGpp, we hypothesize that the induction of the stringent response may provide L/F-transferase access to increased amounts of available aminoacyl-tRNA substrates which otherwise would be sequestered by the translational machinery. Under conditions of a limiting amino acid, the induction of the stringent response alarmone ppGpp would effectively reduce the rate of translation, trapping EF-Tu in various inactive complexes, and, therefore, allowing the accumulation of free aminoacyl-tRNAs for alternative cellular processes, such as the L/F-transferase. Logically, this would make sense to enable the targeting of protein for degradation by L/F-transferase to alleviate the stress of limited amino acids on the cell. If our hypothesis is correct, it would have significant implications on the biological function of L/F-transferase which in general have been elusive. 

While the stringent response can be initiated by the limitation of any amino acid, even the limitation of leucine may also result in the availability of Leu-tRNA^Leu^ (CAG) by this mechanism. As the Leu-tRNA^Leu^ (CAG) isoacceptor is of intermediate sensitivity to leucine as mentioned above, there will be a reduced but significant amount of this highly abundant tRNA^Leu^ (CAG) isoacceptor remaining in the cell upon leucine limitation that could be accessible for L/F-transferase activity.

## 6. Eukaryotic Aminoacyl-tRNA Protein Transferase and tRNA Substrates

As mentioned previously, aminoacyl-tRNA protein transferases are also widely found in eukaryotes [89]. The eukaryotic enzyme (Ate1) utilizes tRNA^Arg^ as a substrate and the resultant protein arginylation leads to a number of different outcomes depending on the substrate protein in question. N-terminal arginylation has long been known to result in proteasome dependent protein degradation for many proteins [89], but additional reports have described the targeting of some proteins for autophagy dependent degradation [90] or stabilization [91,92]. In addition, other examples have been reported that do not alter protein stability but appear to alter the protein function [93,94,95]. What may be unique to ATE1 *versus* the bacterial L/F-transferase is the ability to transfer the amino acid from the tRNA substrate to the side chain of proteins in addition to the N-termini [96,97]. As with the bacterial L/F-transferse, the novel N-terminal addition of amino acids to peptides from aminoacyl-tRNA substrates have also resulted in the proposed use of these enzymes for biochemical applications [98,99].

To date, nothing has been reported regarding tRNA^Arg^ isoacceptor utilization by Ate1, nor is there any insight regarding whether this enzyme must compete for aminoacyl-tRNA substrates with the eukaryotic elongation factors. It is possible that investigations may eventually reveal that there are specialized tRNA^Arg^ isoacceptors in eukaryotes for Ate1. With the high number of genomically encoded tRNA^Arg^ isoaccepors (21 in yeast and 28 in humans) [52], this is an exciting possibility of specialized tRNAs.

## 7. Concluding Remarks

The continued understanding of the utilization of tRNA by the different cellular factors in cells reveals new aspects of the evolutionary constraints on the divergence of tRNA sequences. The recent investigations on the tRNA recognition by L/F-transferase demonstrates a larger set of recognition nucleotides in a tRNA^Leu^ isoacceptor which may constrain sequence divergence. We also pose the question regarding the competition for aminoacyl-tRNAs by the different cellular components, which utilize these molecules for substrates for different biological functions and provide some insights into how this can be circumvented or regulated. While the details regarding this competition for substrates is hypothetical, it does highlight how little we know regarding these small RNAs that have been investigated for over a half a century and have been at the center of a number of major milestones in molecular biology research [100].
